# Ongoing tuberculosis transmission among second-generation Ethiopian immigrants

**DOI:** 10.1017/S0950268820001715

**Published:** 2020-08-03

**Authors:** H. Bishara, M. Green, D. Chemtob, A. Saffouri, L. Zelikman, D. Weiler-Ravell

**Affiliations:** 1 Pulmonary Division and Tuberculosis Center, Galilee Medical Center, Nahariya, and Faculty of Medicine in Galilee, Bar-Ilan University, Safed, Israel; 2 School of Public Health, Faculty of Social Welfare and Health Science, University of Haifa, Haifa, Israel; 3 Department of Tuberculosis & AIDS, Ministry of Health, Jerusalem, Israel; 4 The Hebrew University-Hadassah Medical School, Braun School of Public Health and Community Medicine, Jerusalem, Israel; 5 Tuberculosis Clinic & Internal Medicine, Nazareth Hospital, Nazareth, and Faculty of Medicine in Galilee, Bar-Ilan University, Safed, Israel; 6 Ministry of Health, Yizrael, Afula, Israel

**Keywords:** Epidemiology, infectious disease, public health, Tuberculosis (TB)

## Abstract

Despite considerable efforts to control tuberculosis (TB) among Ethiopian immigrants in Israel, an outbreak of TB among second-generation Ethiopian immigrants that involved native Israelis occurred between January 2011 and December 2019. The aim of this article is to report on this outbreak and discuss the patient and health system barriers that led to its propagation. Overall, 13 culture-positive TB patients were diagnosed in this outbreak. An additional 36 cases with identical mycobacterium tuberculosis genotypes were identified through cross-checking with the National TB Laboratory Registry. Among the 32 close contacts of the index case, 18 (56.3%) reported for screening and treatment of latent TB infection (LTBI) was recommended for 11 (61.1%) of them. However, none completed treatment and eight eventually developed TB. Of the 385 close contacts identified in this outbreak, 286 (74.3%) underwent contact investigation, 154 (53.8%) were recommended LTBI treatment, but only 26 (16.9%) completed the treatment. Routine contact investigation and treatment practice measures failed to contain the cascade of infection and disease, leading to the spread of the infecting strain of TB. This report highlights the challenges to identify the high-risk group and address barriers to care among such a vulnerable population.

## Background

During the last three decades, Ethiopians of Jewish descent immigrated to Israel and subsequently, the second generation of Jewish Ethiopian descendants were born in Israel.

Ethiopian immigrants (EI's) have to undergo screening for active tuberculosis (TB) and latent TB infection (LTBI) while in Ethiopia before their departure to Israel. The screening consists of a tuberculin skin test (TST) and chest X-ray. Those with a positive TST or abnormal chest X-ray are referred upon arrival in Israel to one of the TB clinics for further evaluation and treatment. It is worth noting that all TB-related medical care in Israel is provided exclusively in nine specialist centres (TB clinics) dispersed across the country [1].

This report describes an outbreak of TB among second-generation Ethiopians during 2011–2019, which expanded to involve native Israelis as well. The outbreak started with the diagnosis of the index case in January 2011. The index case was a 20-years-old male, Israeli-born EI descendant. He sought medical care for cough and fatigue that had continued for 4 months. He had smear-positive, culture-positive, bilateral pulmonary TB susceptible to first-line treatment medications. He also had a history of alcohol and illicit substance abuse. Due to his poor condition and slow sputum conversion, he was hospitalised for 3 months in a TB ward. The most recent case was a 17-year-old, a close contact of an active TB patient in this outbreak, diagnosed in December 2019.

## Aims

The aim of this report is to describe the clinical and epidemiologic characteristics of this outbreak and contributing factors for poor compliance to LTBI treatment and to discuss the barriers at the patient and health system levels, particularly for high-risk and vulnerable groups.

## Materials and methods

### Tb case definition

Culture-positive TB cases closely related to this group and with isolates matching the outbreak strain by genotyping were defined as part of this outbreak.

Patients with identical mycobacterium tuberculosis genotypes not closely related to the outbreak, who were identified through cross-matching to the National TB laboratory registry, were defined as part of this cluster.

The species identification was performed by a commercial strip DNA probe test (HAIN Lifescience, Nehren, Germany). All *M. tuberculosis* isolates were characterised by the National Mycobacterium Reference Laboratory as previously reported [2].

### Contact investigation

The contacts underwent standard contact investigation as described previously [3]. They underwent a TST; those with a positive TST result (≥ 5 mm) underwent a standard chest X-ray. Sputum and other specimens were tested for Mycobacterium tuberculosis if indicated. Daily isoniazid treatment was the standard first-line treatment offered for contacts with LTBI and monthly physician follow-up appointments at the TB clinic were scheduled.

## Results

The index case had 32 reported close contacts; five were family members, nine co-workers and 18 friends. He used to meet with his friends in a small basement, where they engaged in smoking illicit substances and drinking alcohol. Only 18 (56.2%) of his close contacts reported for screening; 11 (61.1%) had LTBI and none completed LTBI treatment (Table 1). During 2011–2019, an additional 12 individuals closely related to this group, including two native Israelis, contracted TB (Table 1).

**Table 1.** Epidemiological and clinical characteristics of TB patients

	Age	Gender	Epidemiologic connection	Risk factors	Employment	Diagnosis date	Organ involved	Smear/culture	Creported/C examined	Treatment Indicated Completed	
**IC**	20	Male	IC	DA/Alc	Casual job	January 2011	Lungs	+/+	32/18	11	0
**Pt 1**	27	Male	IC friend	Alc/	Casual job	January 2012	Lungs	+/+	11/8	6	2
**Pt 2**	30	Male	IC friend	DA/Alc	Unemployed	December 2012	Lungs	+/+	9/8	8	3
**Pt 3**	27	Male	IC cousin	Alc,	Unemployed	February 2013	Lungs	−/+	27/20	9	3
**Pt 4**	20	Male	Pt2 brother	Alc,	Army draft	February 2013	Lungs	−/+	23/20	15	6
**Pt 5**	26	Male	IC friend	Alc,	Unemployed	April 2013	Lungs	+/+	28/23	13	2
**Pt 6**	24	Male	Pt1 nephew	DA/Alc,	Casual job	April 2013	Lungs	+/+	17/11	3	1
**Pt 7**	26	Male	Pt1 brother	HIV/Alc,	Unemployed	September 2013	Lungs	+/+	16/16	12	4
**Pt8**	25	Female	Pt2 sister	–	Factory	July 2015	Lungs	+/+	102/85	47	0
**Pt 9#**	22	Female	Pt8 contact	–	Factory	March 2016	Pleura	−/+	0	0	0
**Pt 10**	26	Male	Pt8 contact	DA/Alc	Employed	April 2016	Lungs	+/+	59/49	21	3
**Pt 11#**	25	Male	Pt8 contact	–	Factory	October 2017	Lungs	+/+	18/12	6	0
**Pt 12**	17	Female	Pt8 sister	–	School	December 2019	Lungs	+/+	43/16	3	2

IC, index case; Pt, patient; DA, drug abuse; ALC, alcohol abuse; C, contacts; #, native Israeli.

Of the 385 contacts identified in this outbreak, 286 (74.3%) underwent contact investigation, 154 (53.8%) had a positive TST and 135 (87.7%) started LTBI treatment, however, only 26 (16.9%) completed treatment.

All 13 culture-positive cases were infected with an identical *M. tuberculosis* strain known as Clade T1 SIT 926 (SITVIT database, Institute Pasteur, guadeloupe.fr).

By cross-matching to the National Israeli TB laboratory registry, we identified an additional 36 culture-positive TB cases nationwide infected with this strain. Of those additional 36 culture-positive TB cases infected with this strain nationwide, 33 cases involved first and second-generation immigrants from Ethiopia. The first cases in this cluster were diagnosed in 1998 during a TB outbreak at a boarding school with six culture-positive TB cases among EI in that outbreak [3].

## Discussion

Despite the high treatment initiation rate among TB contacts in this outbreak, only 16.9% eventually completed treatment. This poor outcome led to a cascade of infection and disease that lasted for 9 years.

The current incidence of TB among native Israelis is low (1.0 per 100 000 person-years) and most TB patients are foreign-born [2]. Nevertheless, TB outbreaks in Israel have been reported in congregate settings, where crowded conditions facilitate the spread of TB [3]. However, in such controlled environments, TB outbreaks are short-lived, because the implementation of TB control measures is simple; contacts are easily identified, examined and successfully treated.

We have previously reported high LTBI treatment completion rates (94%) among Ethiopian immigrants in Israel, with directly observed isoniazid treatment and free transportation provided [4]. Those previous findings stand in total contrast to the low LTBI treatment adherence we encountered in this outbreak [3, 4].

There were several barriers to health care, at both the patient and system levels, that may have caused such poor treatment adherence in this outbreak. At the patient level, attending clinic appointments posed challenges, such as the cost and inconvenience of commuting to and from the clinic. Commuting to the TB clinic from the provincial areas of the northern district was challenging for contacts without private transportation. Many of those who missed their follow-up appointment at the TB clinic, declined to return because of the inconvenience of public transportation to the TB clinic. Some individuals involved in this outbreak were unemployed or were employed at low wages. Poverty and financial hardships have been associated with poor treatment adherence [5]. For those who were employed, screening required a day off work to perform a TST and another day off (2 days later), to read the TST reaction. In addition, some were worried that they might lose their jobs for recurrent absence or for being stigmatised as a ‘suspected’ TB patient. This might explain why many contacts who initially presented for screening or LTBI treatment did not return for their scheduled follow-up appointment.

Furthermore, awareness of the possibility of TST cross-reaction with a previous BCG vaccination deterred many contacts from accepting TST ‘positive’ results as indicative of infection [5]. Patient 8 illustrates the pertinence of the obstacles mentioned above. She was a 25-years-old female and an Israeli of EI descent. She was examined in 2012 as a close contact of an active TB patient in this group and isoniazid treatment for LTBI was started. However, she stopped taking her medication after 2 months of treatment and did not report for her scheduled follow-up. She was examined in June 2015 because of a persistent cough and was diagnosed with smear-positive, culture-positive pulmonary TB. She had 102 contacts, mostly co-workers in a factory where she was employed. Of these contacts, 54.9% of those tested were TST positive. Considering the high rate of positive TST in this group, which might in some cases be related to their BCG vaccination and the uncertainties regarding the extent and intensity of exposure, we suggested performing interferon-gamma release assay (IGRA) test to resolve this issue. However, this did not work out and our treatment recommendation was based solely on the TST result. Eventually, these co-workers did not comply with the treatment recommendations. Overall, two of these co-workers contracted active TB.

All 10 male SGE contacts who developed TB in this outbreak abused alcohol or smoked illicit substances. Such high-risk behaviour has been reported among the youth of immigrant population [6]. Unemployment and the diminished parental status that often accompanies immigration have also been associated with high-risk behaviour among youths of immigrant families. Alcohol abuse is associated with higher TB transmission and lower rates of sputum culture conversion [7]. Substance abuse is also related to an increased risk for TB and increased contagiousness [8]. Finally, the stigma of TB is known to influence health-seeking behaviours and treatment adherence [9]. TB primarily affects the poor and is associated with self-stigmatisation as well as a community-based stigmatizing response [9]. Therefore, some patients were reluctant to reveal their contact details or provided incomplete information to hinder the contact tracing process. Patient 2 in this outbreak was a close contact of the index case. However, he was not reported as such and was not included in the index case's contacts investigation. Patient 2 was identified as part of this cluster only after he contracted active TB.

Apart from the barriers to treatment at the patient level described above, there were barriers at a system-level as well. National TB control programmes (NTP) are usually focused on finding and treating active TB. In Israel, the programme grants active TB patients (as well as recent EI's), monetary incentives during the treatment phase. These interventions likely promoted patient compliance [1, 4]. Monetary incentives (e.g. refund or cost paid for transportation) would sometimes be granted for close contacts on a case-by-case basis as well.

With hindsight, this outbreak could have been anticipated. The poor response to the call to be present for the screening and the low adherence to LTBI treatment should have alerted the health care professionals overseeing this outbreak to the risk of evolution of latent TB cases to active disease.

This ongoing TB transmission among second-generation immigrants raised our awareness regarding the need for a proper definition, identification and management of high-risk groups with obstacles to health care. This outbreak illustrates the increased risk for TB transmission among second-generation immigrants, not formally defined as a high-risk group and the need to recognise and act upon the specific obstacles to health care facing this community. Routine contact investigation practices in this outbreak fell short of stopping the cascade of TB transmission and disease, which lasted for 9 years.

## Conclusions

This report describes an ongoing transmission of TB involving second-generation immigrants, which eventually spread to native Israelis. It illustrates the need to recognise and act upon the barriers to health care facing this population. It also raises the issue of defining the high-risk group and the rationale to include second-generation immigrants from high endemic countries. We suggest that individuals with financial hardships or with high-risk behaviour be included so that they would be offered a personalised treatment approach that addresses their health care obstacles and their special needs.

## Data Availability

The data that support the findings of this study are available from the corresponding author (hashemb@gmc.gov.il) with the permission of The Nazareth Hospital.
